# Intrauterine Cataract Diagnosis and Follow-up

**DOI:** 10.4274/tjo.galenos.2020.05014

**Published:** 2020-08-26

**Authors:** Sevinç Aksay, İbrahim Bildirici, Cemile Banu Coşar, Yasemin Alanay, Engin Ciğercioğulları

**Affiliations:** 1 Mersin City Hospital, Clinic of Ophthalmology, Mersin, Turkey; 2 Acıbadem Mehmet Ali Aydınlar University Faculty of Medicine, Department of Obstetrics and Gynecology, Perinatology Unit, İstanbul, Turkey; 3 Acıbadem Mehmet Ali Aydınlar University Faculty of Medicine, Department of Ophthalmology, İstanbul, Turkey; 4 Acıbadem Mehmet Ali Aydınlar University Faculty of Medicine, Department of Pediatrics, Pediatric Genetics Unit, İstanbul, Turkey; 5 Acıbadem Mehmet Ali Aydınlar University Faculty of Medicine, Department of Pathology, İstanbul, Turkey

**Keywords:** Class 3 variant of uncertain significance (c755A>G [P.Lys252Arg]), congenital cataract, autosomal recessive inheritance

## Abstract

In this article, we report a 21-gestational-week fetus diagnosed with congenital cataract by ultrasonography. The parents decided to terminate the pregnancy and asked for examination of the fetus. An amniocentesis was performed for fetal karyotyping. After termination of the pregnancy, fetal autopsy was conducted. Whole exome sequencing (Trio-WES) analysis of the mother and father was done from peripheral blood samples. In the pathologic autopsy report, bilateral anterior and posterior subcapsular cataracts were confirmed. Whole exome sequencing analysis revealed a previously unreported class 3 variant of uncertain significance (c755A>G [P.Lys252Arg]) of the *CRYBB1* gene, which is associated with congenital cataract, that was homozygous in the fetus and heterozygous in the parents. The obtained result is consistent with a genetic diagnosis of isolated autosomal recessive congenital cataract.

## Introduction

Congenital cataract is the most important cause of visual loss in children, and the incidence was reported 1 in 4000 live births.^1^ The etiology of congenital cataract varies, including intrauterine infections, metabolic diseases, and genetic disorders. Cataract can be inherited as an isolated trait (approximately 70% of congenital cataract cases), while 15% of cases are associated with other ocular anomalies such as microcornea and microphthalmia. The remaining 15% of cases are part of a syndrome. The inheritance of most isolated congenital cataracts is autosomal dominant, but autosomal recessive and X-linked forms have also been observed.^2,3,4,5^ To date, 39 genetic mutations have been reported in isolated or primary congenital cataracts, and this number continues to increase. Twenty-six of these mutations are in specific genes. Of the families for whom the mutant gene for congenital cataract is known, about half have mutations in lens-related crystallin and about a quarter have mutations in connexins.^6^ In this case report, we present a fetus diagnosed with congenital cataract in utero.

## Case Report

**Figure 1:**
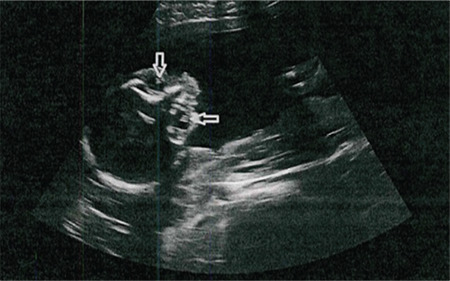
Ultrasound scan image of the fetus in utero at 21 weeks of gestation. Congenital cataracts appear as hyperechoic discs within the orbits (arrows)

A 35-year-old woman, gravida 1 para 0, at 21 weeks’ gestation following *in vitro* fertilization was referred to our eye clinic after detection of bilateral dense, echogenic lenses in the fetus on ultrasonographic examination by a perinatologist (Figure 1). Her medical history was unremarkable in regard to congenital anomalies. She and her husband were second-degree cousins. Maternal hemogram, biochemistry, and microbiologic workup was performed. Amniocentesis was performed for fetal karyotyping and chromosomal microarray analysis. The parents were informed about the treatment and follow-up of congenital cataract and received genetic counseling. Fetal autopsy and next-generation genetic testing were recommended for possible defects which cannot be detected by ultrasonography if the parents decided to terminate the pregnancy. Due to the burden of treatment, with the request of the couple and approval of the Perinatology Council, a medical abortion and autopsy were performed. Whole exome sequencing (trio-WES) analysis was performed using fetal DNA extracted from cultured amniocentesis and parental DNA samples from peripheral blood.

**Figure 2:**
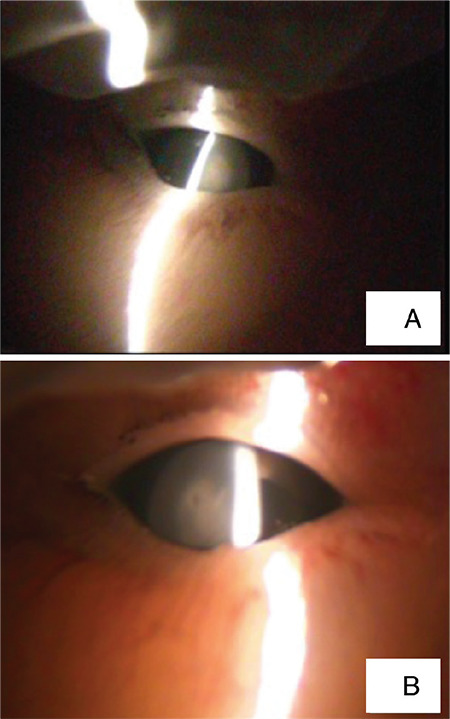
The macroscopic appearance of cataracts in the right eye (A) and in the left eye (B)

Maternal hemogram and biochemistry results were within normal limits. The results of microbiologic workup were as follows: cytomegalovirus (CMV) immunoglobulin (Ig)G antibody positive (165.1 AU/mL), CMV IgM antibody negative (0.13 AU/mL), herpes simplex virus (HSV) type 2 IgG antibody negative (0.1 RU/mL), HSV type 2 IgM antibody negative (0.1), rubella IgG antibody positive (64 IU/mL), rubella IgM antibody negative (0.35 IU/mL), *Toxoplasma* IgG antibody negative (0.1 IU/mL), *Toxoplasma* IgM antibody negative (0.17 IU/mL), Venereal Disease Research Laboratory test negative, and varicella-zoster virus IgG antibody positive (549 mIU/mL). The parents’ ophthalmological examinations were normal and lenses were clear. Ultrasonographic examination of the fetus correlated with the gestational week. Except for bilateral dense lenses, no fetal anomaly was detected. At autopsy, a male fetus consistent with the 20-21 gestational week with broad thumbs and bilateral opaque lenses prediagnosed as cataract and/or persistent hyperplastic primary vitreous was detected macroscopically. The cornea was clear and there was opacity beneath the anterior lens capsule and at the posterior pole (Figure 2A, B). On microscopic examination, bilateral anterior and posterior subcapsular congenital cataracts were reported, while the vitreous was clear. There were no central nervous system findings. Trio-WES revealed a (class 3) variant of uncertain significance (c755A>G [P.Lys252Arg]) in the *CRYBB1* gene, which is associated with congenital cataract, that was homozygous in the fetus and heterozygous in the mother and father. This variant has not been reported in the previous literature.

## Discussion

Cataract is defined as any opacity of the crystalline lens which causes differences in the refractive index of the lens. Congenital cataracts have many clinical and genetic variations. Between 8.3% and 25% of congenital cataracts are believed to be inherited and about half involve mutations in crystallin genes.^6^ Crystallin genes encode more than 95% of the water-soluble proteins present in the vertebrate crystalline lens and are divided into alpha, beta, and gamma subgroups.^7^ The transparency and high refractivity of the normal crystalline lens is dependent on its ability to express crystallin proteins at high concentrations and in a particular spatial arrangement. Mutations in crystallin genes are thought to be the most important cause of inherited congenital cataracts.^4^ In this case, we detected a mutation in the *CRYBB1* gene, which encodes the beta crystallin, which is important in lens transparency and homeostasis. Although congenital cataracts due to *CRYBB1* gene mutations have been reported, mutations in this nucleotide were not previously detected. It is classified as a (class 3) variant of uncertain significance (c755A>G [P.Lys252Arg]). Heterozygous carriage of the variant by the mother and father, who were asymptomatic, and homozygous presence in the fetus is suggestive of autosomal recessive inheritance. The autopsy findings and review of the literature suggest that these findings are consistent with a novel mutation in the *CRYBB1* gene and a genetic diagnosis of isolated autosomal recessive cataract. The *CRYBB1* gene encodes a 252-amino acid protein mainly expressed in the early lens nucleus. Beta B1 crystallin, a major subunit of the beta-crystallins, comprises 9% of the total soluble crystallins in the human lens, and this amount decreases dramatically with age. Beta B1 crystallin is thought to be important for the maintenance of lens transparency.^7^ A mutation was found in the *CRYBB1* gene that caused autosomal dominant pulverulent cataract which was bilateral in all cases, consisted of fine, dust-like opacities that mainly affected the central zone, or fetal nucleus, of the lens but also affected the cortex, anterior and posterior Y-suture regions. Other ocular pathologies and systemic diseases were not seen in the patients.^3^ Another mutation was reported in the *CRYBB1* gene that caused autosomal dominant congenital cataract and microcornea. The cataract seen in the patients was dense and nuclear, and included cortical fibers and anterior and posterior polar opacities.^8^ Another autosomal recessively inherited congenital cataract due to a different mutation in the *CRYBB1* gene was reported. The cataract was bilateral and nuclear in all patients.^4^

During morphogenesis, the lens develops by the formation of an embryonic nucleus. Throughout life, lens fibers are deposited around the embryonic nucleus, forming first the fetal nuclear region and later the cortex. In inherited congenital cataracts, location of the lens opacification gives a clue about the underlying genotype. Nuclear cataract, which is usually bilateral and symmetrical, is common and suggests an early developmental gene expression abnormality. Cataract affecting the cortex is rare. The opacification is usually seen in the outer, superior cortical region, while the nucleus is clear. This type of cataract suggests a late lens developmental abnormality.^2^ In our case, we found anterior and posterior subcapsular cataracts suggesting that the mutant gene affected the fetal nucleus in the late stages of development. Microbiological test results did not reveal an infectious etiology. Although various papers about congenital cataracts in Turkey have been published,^9,10^ our case is the first report of congenital cataracts diagnosed prenatally.

In conclusion, we report a novel mutation in the *CRYBB1* gene causing anterior and posterior subcapsular cataracts, suggesting that the mutant gene affected the fetal nucleus in the late stages of development. The risk for congenital cataract recurrence is 25%. Genetic counseling and prenatal next-generational testing are currently the best practice in the detection of cataracts in utero.
